# Hyperalgesic activity of kisspeptin in mice

**DOI:** 10.1186/1744-8069-7-90

**Published:** 2011-11-23

**Authors:** Simona Spampinato, Angela Trabucco, Antonella Biasiotta, Francesca Biagioni, Giorgio Cruccu, Agata Copani, William H Colledge, Maria Angela Sortino, Ferdinando Nicoletti, Santina Chiechio

**Affiliations:** 1 Department of Clinical and Molecular Biomedicine, University of Catania, Italy; 2 Department of Neuropharmacology, University of Catania, Italy; 3 I.N.M. Neuromed, Pozzilli, Italy; 4 Department of Neurology and Psychiatry, University of Rome "Sapienza", Italy; 5 Department of Drug Sciences, University of Catania, Italy; 6 Department of Physiology, Development, and Neuroscience, University of Cambridge, UK; 7 Department of Physiology and Pharmacology, University of Rome "Sapienza", Italy

**Keywords:** Kisspeptin, GPR54, inflammatory pain, nociceptive sensitization

## Abstract

**Background:**

Kisspeptin is a neuropeptide known for its role in the hypothalamic regulation of the reproductive axis. Following the recent description of kisspeptin and its 7-TM receptor, GPR54, in the dorsal root ganglia and dorsal horns of the spinal cord, we examined the role of kisspeptin in the regulation of pain sensitivity in mice.

**Results:**

Immunofluorescent staining in the mouse skin showed the presence of GPR54 receptors in PGP9.5-positive sensory fibers. Intraplantar injection of kisspeptin (1 or 3 nmol/5 μl) induced a small nocifensive response in naive mice, and lowered thermal pain threshold in the hot plate test. Both intraplantar and intrathecal (0.5 or 1 nmol/3 μl) injection of kisspeptin caused hyperalgesia in the first and second phases of the formalin test, whereas the GPR54 antagonist, p234 (0.1 or 1 nmol), caused a robust analgesia. Intraplantar injection of kisspeptin combined with formalin enhanced TRPV1 phosphorylation at Ser800 at the injection site, and increased ERK1/2 phosphorylation in the ipsilateral dorsal horn as compared to naive mice and mice treated with formalin alone.

**Conclusion:**

These data demonstrate for the first time that kisspeptin regulates pain sensitivity in rodents and suggest that peripheral GPR54 receptors could be targeted by novel drugs in the treatment of inflammatory pain.

## Background

Kisspeptin is a 54-amino acid peptide originally discovered for its activity as metastasis-suppressor [1]. It is encoded by the Kiss1 gene as a 145-amino acid precursor protein and cleaved to a 54-amino acid protein as well as into shorter products (kisspeptin-10,-13,-14) known to play a critical role in the neuroendocrine regulation of reproduction [2-5].

In the brain, kisspeptin is localized not only in areas involved in gonadotropin secretion, but also in other regions such as the amygdala, hippocampus, and the periacqueductal gray [6,7].

Its action is mediated by a 7-TM receptor named GPR54, also known as KISS1R, which is coupled to polyphosphoinositide hydrolysis *via *a G_q/11 _GTP binding protein [2,8].

Loss-of-function mutations of GPR54 cause a non-Kallman variant of hypogonadotropic/hypogonadism in humans (i.e. hypogonadotropic/hypogonadism without anosmia) [2,9]. Interestingly, the expression of kisspeptin and GPR54 is not restricted to the hypothalamus. Relatively high levels of kisspeptin and GPR54 are found in forebrain regions, such as the hippocampus and amygdala, as well as in the periacqueductal grey [10]. The investigation of the extrahypothalamic functions of kisspeptin is still at its infancy. Treatment with kainic acid increases kisspeptin mRNA levels in the hippocampus, and kisspeptin enhances the amplitude of excitatory postsynaptic currents in granule cells of the hippocampal dentate gyrus [6,7]. This suggests a potential role for kisspeptin in the regulation of synaptic plasticity in the CNS. Recent findings have shown an intense kisspeptin and GPR54 immunostaining in dorsal root ganglia (DRG) neurons and in lamina I and II of the dorsal horns of the spinal cord [11,12]. The transcripts of kisspeptin and GPR54 are up-regulated in DRG and dorsal horn neurons in the complete Freund adjuvant (CFA) model of chronic inflammatory pain [12], suggesting that kisspeptin may play a role in mechanisms of nociceptive sensitization. However, how precisely kisspeptin regulates pain sensitivity is obscure at present.

We now report that peripheral or intrathecal injection of kisspeptin causes hyperalgesia and induces biochemical changes that are consistent with mechanisms of peripheral and central nociceptive sensitization.

## Methods

### Animals

Adult male CD1 mice (Charles River, Calco, CO, Italy), 129S6/Sv/Ev wild-type, and 129S6/Sv/Ev *Gpr54- *knock-out mice [13] aged between 8 and 9 weeks were used in these experiments. Mice were housed 10 animals per cage with food and water *ad libitum *in standard 12/12 h light/dark cycle, for a period of 2 weeks before testing.

All experiments were carried out according to the recommendations of Institutional Animal Care and Use Committee (IACUC). All efforts were made to minimize animal suffering and to reduce the number of animals used.

### Drug administration

Kisspeptin (Calbiochem Merck KGaA, Darmstadt, Germany) was dissolved in 5% DMSO and injected intrathecally (3 μl) or subcutaneously (5 μl) into the plantar surface of the right hind paw using a 10 μl luertip-syringe (Hamilton) fitted with a 30-gauge needle. p234 (Sigma-Aldrich, St. Louis, MO) was dissolved in phosphate buffered saline (PBS) and injected in a volume of 3 μl for intrathecal administration or 5 μl for intraplantar administration.

### Behavioral experiments

#### Hot plate test

The hot plate test (Ugo Basile, Italy) was used to asses thermal sensitivity. CD1 mice were placed onto the hot plate at the temperature of 55 ± 0.1°C. Paw withdrawal thresholds were determined in the hind paws of ipsilateral hind limb. Animals were kept on the plate until the first sign of ipsilateral paw lift or lick was recorded as basal withdrawal latency (pre-drug latency). A maximum cut-off paw withdrawal latency of 20 seconds was chosen to prevent tissue damage (cut-off time). Post-dose thresholds were taken at 5, 15, 30, and 60 minutes after drug administration (post-drug latency). For each animal, results were expressed as the percentage maximum possible effect (%MPE) calculated using the following formula: [(post-drug latency - pre-drug latency)/(cut-off time - pre-drug latency)] × 100.

#### Formalin test

Inflammatory pain was assessed using the formalin test. Ten μl of a 5% formalin solution was injected subcutaneously into the plantar surface of the right hind paw of CD1 mice. After the injection, mice were immediately placed in a plexiglas box (20 × 15 × 15 cm) surrounded by mirrors to allow the observation of nociceptive responses that include licking, lifting and shaking of the injected paw. Tests were performed between 08:00 h and 12:00 h to minimize variability. Mice were observed for 1 hour. Formalin scores were separated into two phases, phase I (0-10 min) and phase II (15-45 min). The mean behavioural score was calculated in blocks of 5 min for each of the two phases. A mean response was then calculated for each phase.

#### Spontaneous pain

CD1 mice that received intraplantar injection of kisspeptin or vehicle were placed in a cage immediately after the injection, and the duration of hind paw lifting and licking during the first 5 minutes were measured.

All behavioral tests were analyzed by observers blind to the treatment of the animals.

### Immunohistochemistry

#### Skin biopsies

Animals were euthanized with chloral hydrate (320 mg/kg i.p.). 2.5-mm punch skin biopsies from the plantar surface of the hind paws were performed and fixed in Zamboni fixative (2% paraformaldehide, 15% picric acid saturated aqueous solution, 0.1 M phosphate buffer pH 7.4) for 24 hours. Biopsies were cryoprotected with 20% sucrose in PBS overnight at 4°C. Sections of 10 μm were cut at the cryostat and mounted on glass slides for immunohistochemical analysis.

Immunohistochemistry procedures were performed as previously described [14]. Double immunofluorescence was performed in skin biopsies from CD1 male mice incubating sections overnight with polyclonal rabbit anti-human PGP 9.5 (1:1000; AbD Serotec, Kidlington, UK) and goat polyclonal anti-GPR54 (1:20; Santa Cruz Biotechnology, Santa Cruz, CA) and then for 1 h with secondary fluorescein anti-rabbit (1:100; Vector Laboratories, Burlingame, CA) and Cy3 anti-goat (1:400; Chemicon, Billerica, MA) antibodies. Control staining was performed without the primary antibodies.

Immunostaining was performed in skin biopsies from male 129S6/Sv/Ev wild-type and 129S6/Sv/Ev *Gpr54- *knock-out mice [13] to test the specificity of the anti-GPR54 antibody. Tissue sections were incubated overnight with goat polyclonal anti-GPR54 (1:20; Santa Cruz Biotechnology, Santa Cruz, CA) and then for 1 h with secondary biotin-coupled anti-goat (1:100; Vector Laboratories, Burlingame, CA). SG (SG substrate kit; Vector Laboratories, Burlingame, CA, USA) chromogen was used for detection.

#### Spinal cord

CD1 mice (n = 5 per group) were used. 3 min after kisspeptin (3 nmol) or vehicle (DMSO) were co-injected with formalin in the right hind paw and lumbar spinal cords were removed and fixed in formalin (4%) overnight, transferred in 70% ethanol and included in paraffin. Ten serial sections were cut and used for immunohistochemical analysis. Deparaffinized sections were treated with 10 mmol/L citrate buffer, pH 6.0, and heated by microwave for 10 minutes for antigen unmasking. Sections were soaked in 3% hydrogen peroxide to block endogenous peroxidase activity. Tissue sections were incubated overnight with monoclonal rabbit antibody anti-phospho-p44/42 (Erk1/2) (Thr202/Tyr204) (D13.14.4E)XP™ (1:200; Cell Signaling Technology, Denver, MA, USA) and then for 1 h with secondary biotin-coupled anti-rabbit (1:200; Vector Laboratories). 3,3-Diaminobenzidine tetrachloride was used for detection. Control staining was performed without the primary antibodies.

#### Densitometric analysis of p-ERK immunoreactivity

Intensity of p-ERK immunoreactivity was quantified by measuring the optical densities of the outer laminae of the dorsal horn in the stained sections relative to the background (ventral horn). Images were acquired using a computer-based microdensitometer (NIH Image Software, Bethesda, MD, USA). Values were the mean of measurements made on ten sections (10 μm) sampled 1 into a 3 series spanning the extent of the L4-L5 spinal cord.

### Western blot analysis

CD1 Mice were sacrificed 3 min following treatment and skin lysates of all groups were processed in western blot. Skin homogenates were obtained as previously described [15]. Ten μg of total protein were separated by 10% SDS-polyacrylamide gel electrophoresis and electrophoretically transferred onto protein-sensitive nitrocellulose membranes (Criterion blotter; Bio-Rad Laboratories, Hercules, CA). The membranes were blocked in Odyssey blocker (LI-COR Biosciences, Lincoln, NE) for 1 h, and the following primary antibodies were used: anti-TRPV1 (phospho S800) polyclonal antibody (1:400, Abnova, Aachen, Germany); anti-actin monoclonal antibody (1:1000, Sigma). Secondary antibodies were: goat anti-rabbit (IRD800CW) and goat anti-mouse (Alexa 680, LI-COR, Bioscience) antibodies. Proteins were detected with the Odyssey Infrared Fluorescence Imaging System (LI-COR).

## Results

**Figure 1:**
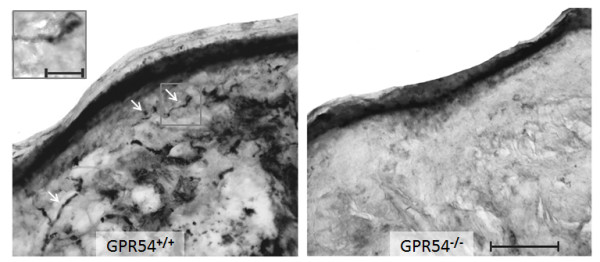
**Immunostaining for the kisspeptin receptor, GPR54, in the mouse skin of GPR54 WT and KO mice**. Representative immunostaining showing the specificity of the GPR54 antibody in the peripheral nerve endings of the mouse skin of GPR54^+/+ ^mice (left panel). No immunostaining is observed in GPR54^-/- ^mice (right panel). Scale bar 100 μm. The insert shows an immunopositive fiber at higher magnification (scale bar = 20 μm).

**Figure 2:**
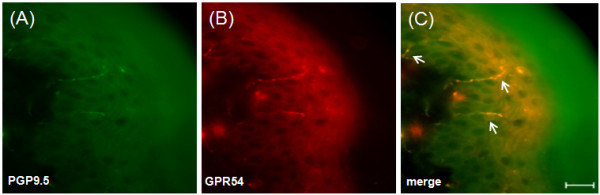
**Double immunofluorescent staining for the kisspeptin receptor, GPR54, and PGP9.5 in the mouse skin**. Immunofluorescent staining of PGP9.5 and GPR54 is shown in (A) and (B), respectively. Co-immunolocalization is shown in (C) (see arrowheads). Scale bar 20 μm.

**Figure 3:**
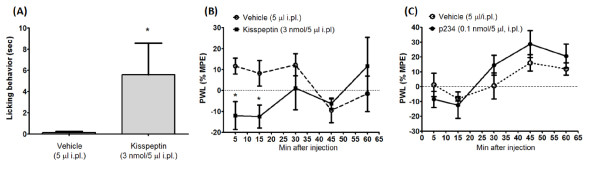
**Intraplantar injection of kisspeptin lowers pain threshold in the hot plate**. The nocifensive response to intraplantar injection of kisspeptin (3 nmol/5 μl) in naïve mice is shown in (A). Data are means ± S.E.M of 6 mice, and refer to the number of sec spent in licking behavior in the first 5 min following injection. *p < 0.05 (Student's t test) vs. mice injected with vehicle. Data obtained in the hot plate test are shown in (B). For each animal, the percentage maximum possible effect (%MPE) was calculated using the following formula: [(post-drug latency) - (pre-drug latency)/(cutoff time) - (pre-drug latency)] × 100. Data are means ± S.E.M. of 6 to 8 mice. *p < 0.05, two-way ANOVA followed by Fisher's *post hoc *test. PWL, Paw-withdrawal latency.

Knowing that the kisspeptin receptor, GPR54 (KISS1R), is present in DRG neurons [12], we performed immunofluorescent analysis to examine whether the receptor was also present in peripheral nociceptors. We focused on the peripheral role of kisspeptin in the modulation of acute and inflammatory pain. First we examined the specificity of the GPR54 antibody in skin biopsies from GPR54 KO mice. No immunostaining was seen in sensory nerve terminals of GPR54 KO mice (Figure 1). The nature of the nonspecific staining seen in the outer portion of the skin of GPR54 KO mice is unknown. In punch skin biopsies from the mouse hind paw, sensory fibers ascending vertically between the keratinocytes to reach the stratum corneum of the epidermis were identified by fluorescent immunostaining for the neuron-specific ubiquitin hydrolase, PGP9.5 [14] (Figure 2A). These fibers also expressed GPR54, as shown by double fluorescence immunostaining (Figure 2B, C). Behavioral experiments were performed after peripheral (intraplantar) and central (intrathecal) administration of kisspeptin at doses ranging from 0.1 to 3 nmol [16]. We first examined the effect of intraplantar injection of kisspeptin on nocifensive behavior in naïve mice. Nocifensive behavior consisting of licking, flinching and shaking of the injected paw was evaluated after a single injection of kisspeptin (3 nmol/5 μl) or vehicle into the plantar surface of the right hind paw. The time spent in nocifensive behavior was recorded for 5 min after the injection. Intraplantar injection of kisspeptin (3 nmol/5 μl) induced brief nocifensive behavior that lasted for about 5-15 seconds, whereas no signs of pain were seen in vehicle-injected mice (Figure 3A). We then assessed the effect of kisspeptin on acute thermal pain using the hot plate test. Intraplantar injection of kisspeptin (3 nmol/5 μl) significantly reduced paw withdrawal latency in response to heat as compared to intraplantar injection of vehicle (Figure 3B), whereas no differences were observed after p234 injection (0.1 nmol/5 μl) (Figure 3C).

**Figure 4:**
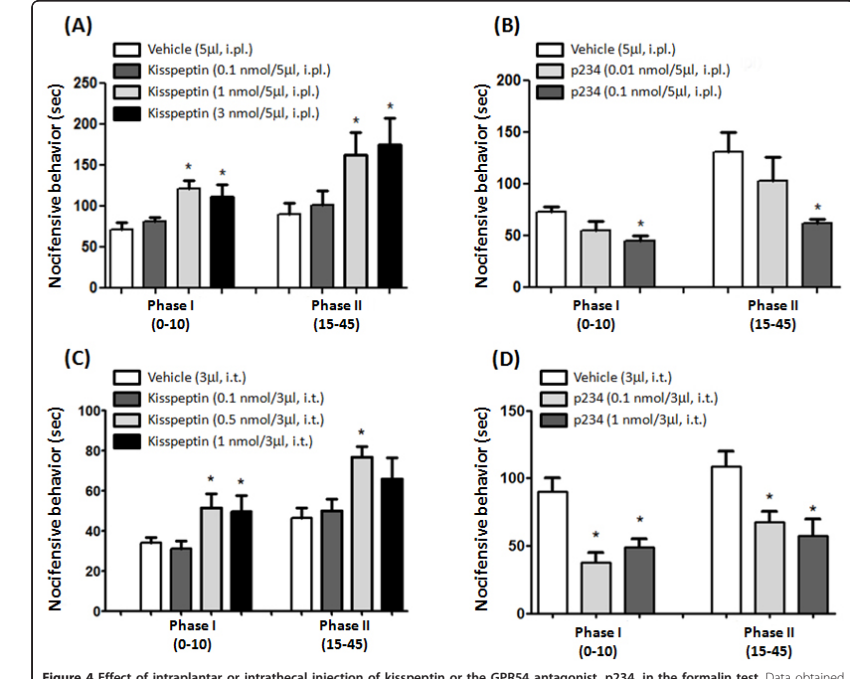
**Effect of intraplantar or intrathecal injection of kisspeptin or the GPR54 antagonist, p234, in the formalin test**. Data obtained with intraplantar (i.pl.) injection of kisspeptin (1 or 3 nmol/5 μl) or p234 (0.01 or 0.1 nmol/5 μl) on the first (0-10 min) and second (15-45 min) phases of the formalin test are shown in (A) and (B), respectively. Drugs were injected 15 min prior to the intraplantar injection of formalin. Data obtained with intrathecal (i.t.) injection of kisspeptin (0.5 or 1 nmol/3 μl) or p234 (0.1 or 1 nmol/3 μl) are shown in (C) and (D), respectively. Data are means + S.E.M. of 8-12 mice per group. *p <0.05 vs. the respective groups of mice injected with vehicle (one-way ANOVA followed by Fisher's *post hoc *test).

**Figure 5:**
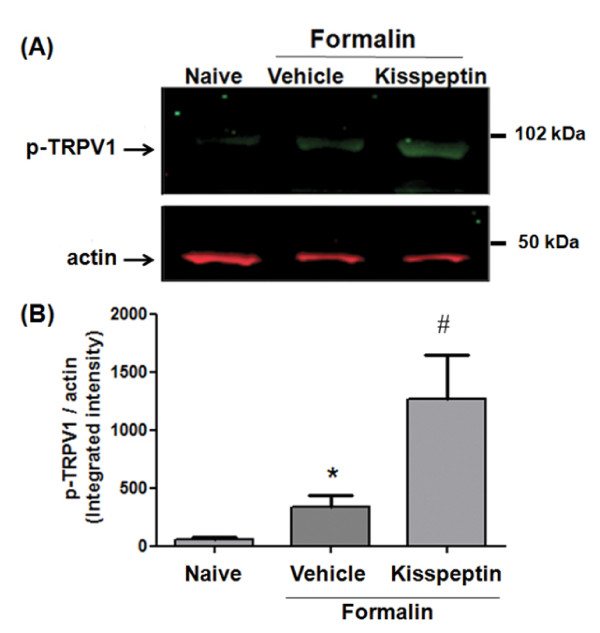
**Intraplantar injection of kisspeptin amplified the increase in TRPV1 phosphorylation in the skin of mice treated with formalin**. A representative immunoblot of (Ser800)-phosphorylated TRPV1 in the skin of naïve mice and mice injected with formalin in the absence or presence of kisspeptin (3 nmol/5 μl) is shown in (A). Densitometric analysis is shown in (B), where values are means + S.E.M. of 4 determinations. *p < 0.05 vs. naïve mice, ^#^p < 0.05 or vs. mice treated with formalin alone (one-way ANOVA followed by Fisher's *post hoc *test).

**Figure 6:**
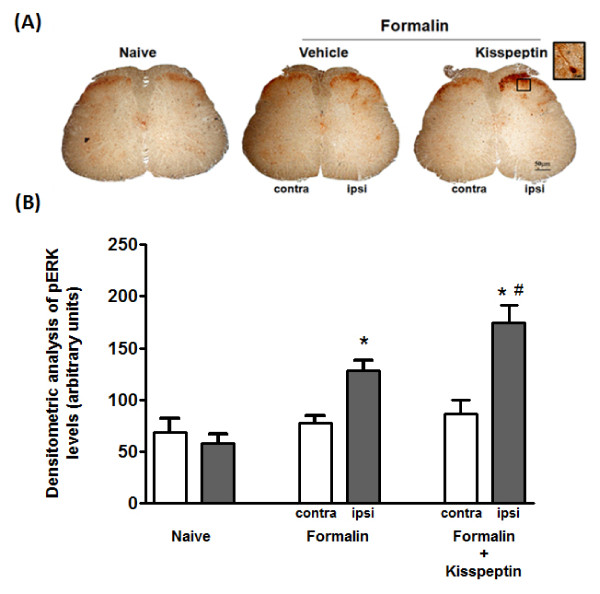
**Intraplantar injection of kisspeptin increased ERK phopshorylation in the ipsilateral dorsal horn of the spinal cord**. (A) Immunohistochemical analysis of phosphorylated-ERK1/2 in the dorsal horns of the spinal cords of naïve mice and mice treated with formalin in the absence or presence of kisspeptin (3 nmol/5 μl) is shown. Contra = contralateral; ipsi = ipsilateral. Scale bar = 50 μm. The insert shows an immunopositive neuron at higher magnification (scale bar = 10 μm). (B) Densitometric analysis of p-ERK immunoreactivity in the superficial laminae of the dorsal horn. *p < 0.05 vs. contralateral values; ^#^*p *< 0.05 vs. formalin alone values (one-way ANOVA + Dunnett's Multiple Comparison Test).

For the assessment of inflammatory pain, mice were subjected to the formalin test, 15 min after intraplantar (0.1, 1 and 3 nmol/5 μl) or intrathecal (0.1, 0.5 and 1 nmol/3 μl) injection of kisspeptin. Intraplantar injection of formalin elicits a biphasic nocifensive response characterized by licking, lifting and shaking of the injected paw. The first phase of the formalin test, starting immediately after formalin injection and lasting for about 10 min, represents a form of acute pain elicited by direct activation of nociceptors. The second phase of the test (occurring approximately 15-45 min after formalin injection) reflects the development of nociceptive sensitization in the dorsal horns of the spinal cord [17,18]. Intraplantar injection of both 1 and 3 nmol/5 μl of kisspeptin (15 min prior to formalin injection) caused hyperalgesia in the first and second phases of the formalin test whereas no effects were observed at the lower dose of 0.1 nmol/5 μl (Figure 4). We also assessed the effect of the selective GPR54 antagonist, peptide 234 (p234) [19] in the formalin test. As opposed to kisspeptin, intraplantar injection of p234 (1 nmol/5 μl; 15 min prior to formalin) significantly reduced nocifensive behavior (Figure 4B). A lower dose of p234 (0.1 nmol/5 μl) induced a trend to an analgesic effect, which was not statistically significant (Figure 4B). We also examined whether intrathecal injection of kisspeptin or p234 could affect nocifensive behavior in the formalin test. Kisspeptin injected intrathecally at the dose of 1 nmol/3 μl, 10 min prior to intraplantar injection of formalin, significantly increased nocifensive behavior in the first and second phases of the formalin test. A lower dose of kisspeptin (1 nmol/3 μl) caused hyperalgesia in the first phase, and a non-significant trend to hyperalgesia in the second phase of the test (Figure 4C). When injected intrathecally, compound p234 was analgesic at doses of 0.1 and 1 nmol/3 μl in both phases of the formalin test (Figure 4D). The hyperalgesic activity of kisspeptin in both phases of the formalin test led us to investigate whether the peptide could induce biochemical changes that were consistent with mechanisms of peripheral and central sensitization. We therefore examined TRPV1 channel phosphorylation in the skin of the hind paw, and activation of ERK1/2 in the dorsal horns of the spinal cord in mice subjected to intraplantar injection of formalin preceded by kisspeptin or vehicle. Immunoblot analysis with anti-phosphorylated TRPV1 antibodies showed a single band at the expected molecular size of 95 kDa. We observed that in mice pretreated with vehicle, intraplantar injection of formalin slightly increased the levels of phosphorylated TRPV1 in the ipsilateral hind paw as compared to naïve mice. This effect was largely amplified in mice pretreated with kisspeptin (3 nmol/5 μl, 15 min prior to formalin injection) (Figure 5). Activation of the mitogen activated protein kinase (MAPK) pathway was examined by immunohistochemical analysis of phosphorylated ERK1/2 in the dorsal horns of the spinal cord after intraplantar injection of formalin preceded by vehicle or kisspeptin. Formalin injection preceded by vehicle slightly enhanced phosphorylated ERK1/2 immunostaining in the dorsal horn ipsilateral to the injection side as compared to the contralateral dorsal horn or the dorsal horns of naïve mice (Figure 6). Pretreatment with kisspeptin (3 nmol/μl) dramatically enhanced the expression of phosphorylated ERK1/2 in the ipsilateral dorsal horn (Figure 6).

## Discussion

These data offer the first demonstration that kisspeptin, a peptide known for its role in the regulation of the hypothalamic-pituitary-gonadal axis, lowers pain threshold and enhances nocifensive behavior in mice. Immunohistochemical analysis showed the presence of the kisspeptin receptor, GPR54, in peripheral sensory fibers, a finding that is consistent with the detection of GPR54 mRNA and protein in DRG neurons [11,12]. The lack of staining in GPR54 KO mice indicates that GPR54 is present in peripheral nociceptors explaining the hyperalgesia caused by intraplantar injection of kisspeptin in the hot plate and formalin test. We wish to highlight that intraplantar kisspeptin induced only a small nocifensive response on its own, suggesting that a main action of kisspeptin is to amplify pain sensitivity in response to noxious stimuli. Intraplantar injection of the GPR54 antagonist, p234, caused a robust analgesia in the formalin test, suggesting that endogenous kisspeptin acts extracellularly to activate GPR54 receptors during inflammatory pain. Kisspeptin is present in DRG neurons, where it co-localizes with isolectin B4 and calcitonin gene-related peptide, and its expression is up-regulated by chronic inflammatory pain [12]. It is likely that kisspeptin is released from peripheral nociceptors in response to noxious stimuli, therefore behaving as an autocrine/paracrine factor to promote peripheral nociceptive sensitization. Whether other cells can produce and secrete kisspeptin during inflammation is unknown at present. Phosphorylation of the TRPV1 ion channel is a key event in mechanisms of peripheral nociceptive sensitization [20-22]. The TRPV1 receptor can be phosphorylated by multiple protein kinases, including protein kinase A, protein kinase C (PKC), calcium/calmodulin-dependent protein kinase II, and SRC [23-34]. In, particular, PKC phosphorylates TRPV1 at Ser-502 and Ser-800, thus amplifying ion channel activity [31,35-37]. Intraplantar kisspeptin caused a robust increase in (Ser800)-TRPV1 phosphorylation, an effect that was likely mediated by the activation of the GPR54 receptor, with ensuing stimulation of inositol phospholipid hydrolysis, diacylglycerol formation, and PKC activation [2,8]. Thus, kisspeptin might act similarly to other hyperalgesic molecules that activate Gq-coupled receptors and phosphorylate TRPV1 channels in peripheral nociceptors, such as bradykinin, group-I mGlu receptor agonists, P2Y2 receptor agonists, EP1 receptor, and prokineticin [28,38-48].

Hyperalgesia by kisspeptin and analgesia by p234 were also seen in the second phase of the formalin test, which reflects the development of central nociceptive sensitization in the dorsal horns of the spinal cord [17,18]. Central nociceptive sensitization is mediated by a series of mechanisms that ultimately lead to an enhancement of excitatory transmission at the synapses between primary afferent fibers and second order sensory neurons in the dorsal horns of the spinal cord [24]. The relevance of the MAPK pathway in the development of central sensitization has been highlighted in a recent review [48]. Intraplantar injection of formalin is known to induce a rapid phosphorylation of ERK1/2 in the spinal cord, which has been causally related to the increase in nocifensive behavior seen in the second phase of the formalin test [49]. Pharmacological activation of mGlu1 and mGlu5 receptors, which also couple to the Gq protein just like GPR54 [50], can also enhance ERK1/2 phosphorylation in the spinal cord [51]. Activation of GPR54 by kisspeptin has been shown to stimulate the ERK/MAPK pathway both in recombinant expression systems and hypothalamic explants [52,53]. Intraplantar injection of kisspeptin markedly amplified ERK1/2 phosphorylation induced by formalin in the ipsilateral dorsal horn, evidence that nicely supports the behavioral data obtained with kisspeptin in the second phase of the formalin test. Interestingly, kisspeptin retained the hyperalgesic activity (and p234 the analgesic activity) when injected by the intrathecal route. Thus, it is likely that the modulation of pain sensitivity by GPR54 extends beyond peripheral nociceptors. Effects of kisspeptin on different receptors cannot be excluded. In particular it has been reported that kisspeptin can also bind neuropeptide FF (NPFF) receptors [54]. However in our hands intrathecal injection of kisspeptin lowers pain threshold, whereas intrathecal injection of NPFF is known to cause analgesia [55], thus the effect of kisspeptin in the spinal cord is likely mediated by the activation of the GPR54 receptor excluding an interaction of kisspeptin with NPFF receptors.

The presence of GPR54 receptor in the amygdala [56] may suggest that kisspeptin acts also at higher brain centers that control the affective components of pain and contributes to the top-down regulation of pain threshold.

## Conclusions

In conclusion, our data disclose a new aspect in the physiology of kisspeptin and suggest that peripheral GPR54 receptor antagonists (lacking potential hypothalamic side effects) can be developed as new drugs for the treatment of inflammatory pain. In addition, it will be interesting to explore whether individuals with hypogonadotropic hypogonadism due to inactivating mutations of GPR54 show alterations in the sensitivity to pain.

## Competing interests

The authors declare that they have no competing interests.

## Authors' contributions

The study was conceived and the experiments were designed by FN, SC, MAS, AC GC and WHC. SC and SS performed behavioral experiments and western blot analysis. AT, AB and FB, performed immunohistochemical analysis. All authors contributed to writing the manuscript, and all read and approved the final manuscript.

## References

[B1] Lee JH Miele ME Hicks DJ Phillips KK Trent JM Weissman BE Welch DR KiSS-1, a novel human malignant melanoma metastasis-suppressor gene J Natl Cancer Inst 1996 88 1731 1737 10.1093/jnci/88.23.1731 8944003

[B2] Seminara SB Mechanisms of Disease: the first kiss-a crucial role for kisspeptin-1 and its receptor, G-protein-coupled receptor 54, in puberty and reproduction Nat Clin Pract Endocrinol Metab 2006 2 328 334 10.1038/ncpendmet0139 16932310

[B3] Kauffman AS Clifton DK Steiner RA Emerging ideas about kisspeptin- GPR54 signaling in the neuroendocrine regulation of reproduction Trends Neurosci 2007 30 504 511 10.1016/j.tins.2007.08.001 17904653

[B4] Colledge WH Kisspeptins and GnRH neuronal signalling Trends Endocrinol Metab 2009 20 115 21 10.1016/j.tem.2008.10.005 19097915

[B5] Lehman MN Coolen LM Goodman RL Minireview: kisspeptin/neurokinin B/dynorphin (KNDy) cells of the arcuate nucleus: a central node in the control of gonadotropin-releasing hormone secretion Endocrinology 2010 151 3479 3489 10.1210/en.2010-0022 20501670 PMC2940527

[B6] Arai AC Orwig N Factors that regulate KiSS1 gene expression in the hippocampus Brain Res 2008 1243 10 8 18834866 10.1016/j.brainres.2008.09.031

[B7] Arai AC The role of kisspeptin and GPR54 in the hippocampus Peptides 2009 30 16 25 10.1016/j.peptides.2008.07.023 18765263

[B8] Gottsch ML Clifton DK Steiner RA Kisspepeptin-GPR54 signaling in the neuroendocrine reproductive axis Mol Cell Endocrinol 2006 254-255 91 6 16762492 10.1016/j.mce.2006.04.030

[B9] de Roux N Genin E Carel JC Matsuda F Chaussain JL Milgrom E Hypogonadotropic hypogonadism due to loss of function of the KiSS1-derived peptide receptor GPR54 Proc Natl Acad Sci USA 2003 100 10972 10976 10.1073/pnas.1834399100 12944565 PMC196911

[B10] Oakley AE Clifton DK Steiner RA Kisspeptin signaling in the brain Endocr Rev 2009 30 713 743 10.1210/er.2009-0005 19770291 PMC2761114

[B11] Dun SL Brailoiu GC Parsons A Yang J Zeng Q Chen X Chang JK Dun NJ Metastin-like immunoreactivity in the rat medulla oblongata and spinal cord Neurosci Lett 2003 335 197 201 10.1016/S0304-3940(02)01191-6 12531466

[B12] Mi WL Mao-Ying QL Liu Q Wang XW Li X Wang YQ Wu GC The distribution of kisspeptin and its receptor GPR54 in rat dorsal root ganglion and up-regulation of its expression after CFA injection Brain Res Bull 2009 78 254 260 10.1016/j.brainresbull.2008.12.003 19111911

[B13] Seminara SB Messager S Chatzidaki EE Thresher RR Acierno JS Shagoury JK Bo-Abbas Y Kuohung W Schwinof KM Hendrick AG Zahn D Dixon J Kaiser UB Slaugenhaupt SA Gusella JF O'Rahilly S Carlton MB Crowley WF Aparicio SA Colledge WH The GPR54 gene as a regulator of puberty N Engl J Med 2003 349 1614 1627 10.1056/NEJMoa035322 14573733

[B14] McCarthy BG Hsieh ST Stocks A Hauer P Macko C Cornblath DR Griffin JW McArthur JC Cutaneous innervation in sensory neuropathies: evaluation by skin biopsy Neurology 1995 45 1848 1855 7477980 10.1212/wnl.45.10.1848

[B15] Jin L Miyamoto O Toyoshima T Kobayashi R Murakami TH Itano T Localization of calbindin-D28k in normal and incised mouse skin: immunohistochemical and immunoblot analysis Arch Dermatol Res 1997 289 578 84 10.1007/s004030050243 9373717

[B16] Pheng V Uenoyama Y Homma T Inamoto Y Takase K Yoshizawa-Kumagaye K Isaka S Watanabe TX Ohkura S Tomikawa J Maeda K Tsukamura H Potencies of centrally- or peripherally-injected full-length kisspeptin or its C-terminal decapeptide on LH release in intact male rats J Reprod Dev 2009 55 378 82 10.1262/jrd.20240 19384054

[B17] Coderre TJ Melzack R The contribution of excitatory amino acids to central sensitization and persistent nociception after formalin-induced tissue injury J Neurosci 1992 12 3665 3670 1326610 10.1523/JNEUROSCI.12-09-03665.1992 PMC6575737

[B18] Tjølsen A Berge OG Hunskaar S Rosland JH Hole K The formalin test: an evaluation of the method Pain 1992 51 5 17 10.1016/0304-3959(92)90003-T 1454405

[B19] Roseweir AK Kauffman AS Smith JT Guerriero KA Morgan K Pielecka-Fortuna J Pineda R Gottsch ML Tena-Sempere M Moenter SM Terasawa E Clarke IJ Steiner RA Millar RP Discovery of potent kisspeptin antagonists delineate physiological mechanisms of gonadotropin regulation J Neurosci 2009 29 3920 9 10.1523/JNEUROSCI.5740-08.2009 19321788 PMC3035813

[B20] Hucho T Levine JD Signaling pathways in sensitization: toward a nociceptor cell biology Neuron 2007 55 365 76 10.1016/j.neuron.2007.07.008 17678851

[B21] Stucky CL Dubin AE Jeske NA Malin SA McKemy DD Story GM Roles of transient receptor potential channels in pain Brain Res Rev 2009 60 2 23 10.1016/j.brainresrev.2008.12.018 19203589 PMC2683630

[B22] Studer M McNaughton PA Modulation of single-channel properties of TRPV1 by phosphorylation J Physiol 2010 588 3743 3756 10.1113/jphysiol.2010.190611 20693293 PMC2998224

[B23] Tominaga M Caterina MJ Malmberg AB Rosen TA Gilbert H Skinner K Raumann BE Basbaum AI Julius D The cloned capsaicin receptor integrates multiple pain-producing stimuli Neuron 1998 21 531 543 10.1016/S0896-6273(00)80564-4 9768840

[B24] Tominaga M Wada M Masu M Potentiation of capsaicin receptor activity by metabotropic ATP receptors as a possible mechanism for ATP-evoked pain and hyperalgesia Proc Natl Acad Sci USA 2001 98 6951 6956 10.1073/pnas.111025298 11371611 PMC34459

[B25] Premkumar LS Ahern GP Induction of vanilloid receptor channel activity by protein kinase C Nature 2000 408 985 990 10.1038/35050121 11140687

[B26] De Petrocellis L Harrison S Bisogno T Tognetto M Brandi I Smith GD Creminon C Davis JB Geppetti P Di Marzo V The vanilloid receptor (VR1)-mediated effects of anandamide are potently enhanced by the cAMP-dependent protein kinase J Neurochem 2001 77 1660 1663 10.1046/j.1471-4159.2001.00406.x 11413249

[B27] Bhave G Zhu W Wang H Brasier DJ Oxford GS Gereau RW cAMP-dependent protein kinase regulates desensitization of the capsaicin receptor (VR1) by direct phosphorylation Neuron 2002 35 721 731 10.1016/S0896-6273(02)00802-4 12194871

[B28] Hu HJ Bhave G Gereau RW Prostaglandin and protein kinase A-dependent modulation of vanilloid receptor function by metabotropic glutamate receptor5: potential mechanism for thermal hyperalgesia J Neurosci 2002 22 7444 7452 12196566 10.1523/JNEUROSCI.22-17-07444.2002 PMC6757997

[B29] Rathee PK Distler C Obreja O Neuhuber W Wang GK Wang SY Nau C Kress M PKA/AKAP/VR-1 module: A common link of Gs-mediated signaling to thermal hyperalgesia J Neurosci 2002 22 4740 4745 12040081 10.1523/JNEUROSCI.22-11-04740.2002 PMC6758778

[B30] Sugiura T Tominaga M Katsuya H Mizumura K Bradykinin lowers the threshold temperature for heat activation of vanilloid receptor1 J Neurophysiol 2002 88 544 548 12091579 10.1152/jn.2002.88.1.544

[B31] Bhave G Hu HJ Glauner KS Zhu W Wang H Brasier DJ Oxford GS Gereau RW Protein kinase C phosphorylation sensitizes but does not activate the capsaicin receptor transient receptor potential vanilloid 1 (TRPV1) Proc Natl Acad Sci USA 2003 100 12480 12485 10.1073/pnas.2032100100 14523239 PMC218783

[B32] Dai Y Moriyama T Higashi T Togashi K Kobayashi K Yamanaka H Tominaga M Noguchi K Proteinase-activated receptor 2-mediated potentiation of transient receptor potential vanilloid subfamily 1 activity reveals a mechanism for proteinase-induced inflammatory pain J Neurosci 2004 24 4293 4299 10.1523/JNEUROSCI.0454-04.2004 15128843 PMC6729433

[B33] Jung J Shin JS Lee SY Hwang SW Koo J Cho H Oh U Phosphorylation of vanilloid receptor1 by Ca2+/calmodulin-dependent kinase II regulates its vanilloid binding J Biol Chem 2004 279 7048 7054 14630912 10.1074/jbc.M311448200

[B34] Jin X Morsy N Winston J Pasricha PJ Garrett K Akbarali HI Modulation of TRPV1 by nonreceptor tyrosine kinase, c-Src kinase Am J Physiol Cell Physiol 2004 287 C558 563 10.1152/ajpcell.00113.2004 15084474

[B35] Numazaki M Tominaga T Toyooka H Tominaga M Direct phosphorylation of capsaicin receptor VR1 by protein kinase C epsilon and identification of two target serine residues J Biol Chem 2002 277 13375 13378 10.1074/jbc.C200104200 11884385

[B36] Numazaki M Tominaga M Nociception and TRP channels Curr Drug Targets CNS Neurol Disord 2004 3 479 485 10.2174/1568007043336789 15578965

[B37] Mandadi S Tominaga T Numazaki M Murayama N Saito N Armati PJ Roufogalis BD Tominaga M Increased sensitivity of desensitized TRPV1 by PMA occurs through PKCepsilon-mediated phosphorylation at S800 Pain 2006 123 106 116 10.1016/j.pain.2006.02.016 16564619

[B38] Bhave G Karim F Carlton SM Gereau RW Peripheral group I metabotropic glutamate receptors modulate nociception in mice Nat Neurosci 2001 4 417 23 10.1038/86075 11276233

[B39] Hu HJ Alter BJ Carrasquillo Y Qiu CS Gereau RW Metabotropic glutamate receptor 5 modulates nociceptive plasticity via extracellular signal-regulated kinase-Kv4.2 signaling in spinal cord dorsal horn neurons J Neurosci 2007 27 13181 91 10.1523/JNEUROSCI.0269-07.2007 18045912 PMC6673402

[B40] Moriyama T Iida T Kobayashi K Higashi T Fukuoka T Tsumura H Leon C Suzuki N Inoue K Gachet C Noguchi K Tominaga M Possible involvement of P2Y2 metabotropic receptors in ATP-induced transient receptor potential vanilloid receptor 1-mediated thermal hypersensitivity J Neurosci 2003 23 6058 6062 12853424 10.1523/JNEUROSCI.23-14-06058.2003 PMC6740351

[B41] Ferreira J da Silva GL Calixto JB Contribution of vanilloid receptors to the overt nociception induced by B2 kinin receptor activation in mice Br J Pharmacol 2004 141 787 94 10.1038/sj.bjp.0705546 14967737 PMC1574249

[B42] Vellani V Colucci M Lattanzi R Giannini E Negri L Melchiorri P McNaughton PA Sensitization of transient receptor potential vanilloid 1 by the prokineticin receptor agonist Bv8 J Neurosci 2006 26 5109 5116 10.1523/JNEUROSCI.3870-05.2006 16687502 PMC6674238

[B43] Negri L Lattanzi R Giannini E Colucci M Margheriti F Melchiorri P Vellani V Tian H De Felice M Porreca F Impaired nociception and inflammatory pain sensation in mice lacking the prokineticin receptor PKR1: focus on interaction between PKR1 and the capsaicin receptor TRPV1 in pain behavior J Neurosci 2006 26 6716 27 10.1523/JNEUROSCI.5403-05.2006 16793879 PMC6673825

[B44] Malin SA Davis BM Koerber HR Reynolds IJ Albers KM Molliver DC Thermal nociception and TRPV1 function are attenuated in mice lacking the nucleotide receptor P2Y2 Pain 2008 138 484 96 10.1016/j.pain.2008.01.026 18343036 PMC2630699

[B45] Kim YH Park CK Back SK Lee CJ Hwang SJ Bae YC Na HS Kim JS Jung SJ Oh SB Membrane-delimited coupling of TRPV1 and mGluR5 on presynaptic terminals of nociceptive neurons J Neurosci 2009 29 10000 10009 10.1523/JNEUROSCI.5030-08.2009 19675234 PMC6664976

[B46] Mizumura K Sugiura T Katanosaka K Banik RK Kozaki Y Excitation and sensitization of nociceptors by bradykinin: what do we know? Exp Brain Res 2009 196 53 65 10.1007/s00221-009-1814-5 19396590

[B47] Moriyama T Higashi T Togashi K Iida T Segi E Sugimoto Y Tominaga T Narumiya S Tominaga M Sensitization of TRPV1 by EP1 and IP reveals peripheral nociceptive mechanism of prostaglandins Mol Pain 2005 1 3 10.1186/1744-8069-1-3 15813989 PMC1074353

[B48] Ji RR Gereau RW Malcangio M Strichartz GR MAP kinase and pain Brain Res Rev 2009 60 135 148 10.1016/j.brainresrev.2008.12.011 19150373 PMC2666786

[B49] Karim F Bhave G Gereau RW Metabotropic glutamate receptors on peripheral sensory neuron terminals as targets for the development of novel analgesics Mol Psychiatry 2001 6 615 617 10.1038/sj.mp.4000961 11673787

[B50] Nicoletti F Bockaert J Collingridge GL Conn PJ Ferraguti F Schoepp DD Wroblewski JT Pin JP Metabotropic glutamate receptors: From the workbench to the bedside Neuropharmacology 2011 60 1017 41 10.1016/j.neuropharm.2010.10.022 21036182 PMC3787883

[B51] Karim F Wang CC Gereau RW Metabotropic glutamate receptor subtypes 1 and 5 are activators of extracellular signal-regulated kinase signaling required for inflammatory pain in mice J Neurosci 2001 1-21 3771 3779 10.1523/JNEUROSCI.21-11-03771.2001 PMC6762705 11356865

[B52] Kotani M Detheux M Vandenbogaerde A Communi D Vanderwinden JM Le Poul E Brézillon S Tyldesley R Suarez-Huerta N Vandeput F Blanpain C Schiffmann SN Vassart G Parmentier M The metastasis suppressor gene KiSS-1 encodes kisspeptins, the natural ligands of the orphan G protein-coupled receptor GPR54 J Biol Chem 2001 276 34631 34636 10.1074/jbc.M104847200 11457843

[B53] Castellano JM Navarro VM Fernández-Fernández R Castaño JP Malagón MM Aguilar E Dieguez C Magni P Pinilla L Tena-Sempere M Ontogeny and mechanisms of action for the stimulatory effect of kisspeptin on gonadotropin-releasing hormone system of the rat Mol Cell Endocrinol 2006 257-258 75 83 16930819 10.1016/j.mce.2006.07.002

[B54] Oishi S Misu R Tomita K Setsuda S Masuda R Ohno H Naniwa Y Ieda N Inoue N Ohkura S Uenoyama Y Tsukamura H Maeda K Hirasawa A Tsujimoto G Fujii N Activation of Neuropeptide FF Receptors by Kisspeptin Receptor Ligands ACS Med Chem Lett 2011 2 53 57 10.1021/ml1002053 PMC4018085 24900254

[B55] Roumy M Zajac JM Neuropeptide FF, pain and analgesia Eur J Pharmacol 1998 345 1 11 10.1016/S0014-2999(97)01604-X 9593588

[B56] Lee DK Nguyen T O'Neill GP Cheng R Liu Y Howard AD Coulombe N Tan CP Tang-Nguyen AT George SR O'Dowd BF Discovery of a receptor related to the galanin receptors FEBS Lett 1999 446 1 103 7 10.1016/S0014-5793(99)00009-5 10100623

